# Quality of Life Assessment in Students from Polish Universities during the COVID-19 Pandemic According to WHO Quality of Life Questionnaire

**DOI:** 10.3390/ijerph19138117

**Published:** 2022-07-01

**Authors:** Agata Trzcionka, Marta Włodarczyk-Sielicka, Piotr Surmiak, Anna Szymańska, Artur Pohl, Marta Tanasiewicz

**Affiliations:** 1 Department of Conservative Dentistry with Endodontics, Faculty of Medical Sciences in Zabrze, Medical University of Silesia, plac Akademicki 17, 41-902 Bytom, Poland; martatanasiewicz@sum.edu.pl; 2 Department of Geoinformatics and Hydrography, Maritime University of Szczecin, Waly Chrobrego 1-2, 70-500 Szczecin, Poland; m.wlodarczyk@am.szczecin.pl; 3 Department of Neonatology, Faculty of Medical Sciences in Katowice, Medical University of Silesia, ul. Medyków 4, 40-152 Katowice, Poland; piotrek.surmiak@gmail.com; 4 Department of Neonatal Intensive Care, Faculty of Medical Sciences in Zabrze, Medical University of Silesia in Katowice, ul.3-go Maja 13-15, 41-800 Zabrze, Poland; aszymanska@sum.edu.pl; 5 Department of Non-European Political Studies in Poznań, Adam Mickiewicz University, ul. Wieniawskiego 1, 61-712 Poznań, Poland; artur.pohl@amu.edu.pl

**Keywords:** quality of life, COVID-19, WHOQOL-BREF, students

## Abstract

The outbreak of COVID-19 in December 2019 in China influenced the lives of people all over the world. Many had to face the completely new situation of lockdown. These changes influenced many aspects of life. Students’ quality of life changed as well. The aim of the study was to assess the differences in the quality of life of students with regard to the field of study and the knowledge regarding medicine. The study population consisted of 500 students from three Polish universities (Medical University of Silesia, Maritime University of Szczecin and Adam Mickiewicz University, Poznań). Study participants were invited to fill in an online cross-sectional quality of life questionnaire (WHOQOL-BREF) created by the World Health Organization (WHO). The analysis was done using the IBM SPSS Statistics 25.0 programme. The obtained results showed differences in respondents’ reactions in two domains. The lowest resistance to the critical situation was observed in women who studied at the technical university. Higher values of resistance were observed in women studying medical sciences.

## 1. Introduction

The outbreak of COVID-19 in December 2019 in China influenced the lives of people all over the world. The coronavirus causing a severe pneumonia rapidly spread and it soon turned out that the epidemic was no longer a local problem but a pandemic [1]. By 2 August 2020, there were 198,022,041 confirmed cases of COVID-19, and 4,223,460 deaths all over the world [2]. Many countries, including Poland, having to face a completely unknown new threat, decided to undertake a number of precautions. It was recommended to impose strict preventive measures that included: social distancing, staying at home if possible, limitations in social and cultural life (closure of restaurants, cinemas, theaters, gyms), recommendation of e-learning not only for grammar or high schools but also for universities and many other institutions [3]. All these restrictions undoubtedly influenced people’s quality of life. In literature we can find reports of an increase in the number of patients diagnosed with depression, anxiety or insomnia [4]. People were exposed to uncertainty and isolation; very often they complained of a sense of “loss” [5]. Many studies reported high levels of anxiety among students (regarding disruption in daily routine and social relations [6]. In the available literature, it was observed that students’ anxiety was affecting their health [7]. The increase in the usage of psychoactive substances and alcohol was also observed. The most frequently used substances were alcohol, tobacco, marijuana, and cocaine [5].

WHO defines quality of life as an individual’s perception of their position in life in the context of the culture and value systems in which they live and in relation to their goals, expectations, standards and concerns [8]. Factors that influence the quality of life are the ability to live a normal life, ability to adjust, psychological well-being, and functioning in social groups [9]. Despite these definitions of the quality of life, the WHOQOL-BREF specifies the areas of life that needed to be considered in assessments. These areas are: psychological and physical well-being; independence from others; social relations; self beliefs; and their influence on the environment in which the person lives [10]. The WHOQOL-BREF questionnaire is a cross-culturally comparable quality of life measure. The self-report questionnaire contains four domains of quality of life: Physical health (7 items i.e., Q3, Q4, Q10, Q15, Q16, Q17, Q18); psychological health (6 items i.e., Q5, Q6, Q7, Q11, Q19, Q26); social relationships (3 items i.e., Q20, Q21, Q22); and environment (8 items i.e., Q8, Q9, Q12, Q13, Q14, Q23, Q24, Q25). Two other items (Q1, Q2) measure overall quality of life and personal health. Items are rated on a 5-point Likert scale, and each raw domain score is then transformed to a scale ranging from 0 to 100 (in order to make domain scores comparable with the scores used in the WHOQOL-100), with a higher score indicating a higher quality of life. Many authors have used the WHOQOL-BREF scale to investigate the issue of quality of life during the COVID 19 pandemic [11,12,13]. The universality, conciseness, validity and additionally available research results that may be referred to in discussion prompted the authors to use the proposed tool in their own research. The WHOQOL-BREF questionnaire is a new research tool, and is a short version of the WHOQOL-100, recommended for use in case of time restrictions; where respondent burdens must be minimized; and where there is no necessity for detailed answers [8]. It is composed of 26 questions that enable the assessment of four domains of life, its quality and health. Wołowicka and Jaracz adapted the tool to be used in Poland [9]. The Polish version of the WHOQOL-BREF questionnaire had satisfactory reliability and validity for assessing the quality of life of students from three Polish universities. The psychometric properties of the WHOQOL-BREF scale were evaluated by numerous studies in the general population [14,15] and in different clinical populations [4,16], but very rarely in medical students [16]. The daily routine is important to everyone, but particularly to students in their psychological and emotional development. This is why the authors decided to analyze the quality of life (in the critical situation of a global pandemic) of third year students with regard to their sex and field of study.

The aim of the study was to assess the differences in quality of life of students from various faculties with regard to their level of medical knowledge.

## 2. Materials and Methods

The cross-sectional, quantitative, exploratory and descriptive study was realized by Google survey according to the WHO Quality of Life Questionnaire. Data were collected through a questionnaire and analyzed through descriptive statistics.

The study population consisted of 500 students from three Polish universities (randomly selected, simultaneous groups of 100 people, representing different disciplines, including medical) who studied in the 3rd year (Table 1) during the academic study year 2020/2021.

The criteria for the involvement of students in this study were the course of study, voluntary informed written consent to participate, and filling out all items on the WHOQOL-BREF. Study participants were invited to fill in an online cross-sectional quality of life questionnaire (WHOQOL-BREF) created by the WHO (the permission to use the questionnaire was obtained in correspondence with the author). The questionnaire was available in an on-line version (2 weeks, second wave of Coronavirus pandemic in Poland). In the first part of the research, students were asked questions regarding their age, sex and field of study. The second part consisted of 26 questions [8]. Participation in the study was not obligatory. Anonymity when gathering the data was maintained. The approval of the Bioethics Committee of Silesian Medical University was obtained.

### 2.1. Data Collection

Online survey data were collected from 6 November 2020 to 21 November 2020 with students from three Polish universities. This period fully corresponded to the lockdown due to the second wave of COVID-19 in Poland, and students were experiencing the consequences of social isolation and on-line learning. The participants were recruited through a Google survey. Students were contacted and given all the information about the study, and they were asked their participation on a voluntary basis. All the participants were fully informed about the aims of the study and about the confidentiality of the data, and they were also assured that the data would be used only for the purpose of the research and that a refusal to participate would not affect their current and future course of study in any way. Students did not receive any compensation, nor were they motivated in any other way to participate in the research. At any moment, respondents could leave the research without any consequences. Completing the questionnaire took about 10 (±5) min. Questionnaires that were not fully completed were not included in the analysis (all questions had to be answered). Inclusion criteria were as follows: students who gave the informed consent and who were taught by the researchers. Exclusion criteria: no informed consent, or not filling in the questionnaire completely. Special precautions were provided in order to protect the personal data of study participants. There were 500 students who voluntarily took part in the research and completed the questionnaires.

### 2.2. Statistical Analysis

The analysis was done with the usage of the IBM SPSS Statistics 25.0 programme (IBM Corp., Armonk, NY, USA). Basic descriptive statistics were calculated with the Kolmogorov-Smirnov test. The Cronbach’s alpha coefficient was used to assess reliability. Then the two-variable analysis was done in order to assess the differences in quality of life with regard to sex and field of study. Statistical significance level was α = 0.05.

## 3. Results

In the first stage of the analysis the basic descriptive statistics with the normality of distribution test by Kolmogorov-Smirnov and with the improvement of Lilliefors were calculated. The results proved that none of the analyzed domains was of normal distribution, but taking into consideration sample size and skew values from −2.0 to 2.0, it may be assumed that asymmetry of the results was not significant [11]. The level of internal consistency for the items of the WHOQOL-BREF instrument was measured using the Cronbach’s α coefficient, which was 0.762 for the physical health domain, 0.850 for the psychological health domain, 0.675 for the social relationships domain, and 0.758 for the environment domain. Cronbach’s alpha ranges from r = 0 to 1, with r = 0.7 or greater considered as sufficiently reliable. In accordance to the alpha Cronbach test it was confirmed that three out of four domains were characterized with satisfactory reliability (physical, physiological and environmental), the fourth (social) was characterized with a lower level of reliability but still acceptable [12]. The results of the Cronbach analysis are presented in Table 2.

### 3.1. Sex and the Field of Study and the Quality of Life

In order to check if sex and the field of study changes the quality of life in particular domains the two-factor analysis of variances in scheme 2 × 5 was done.

### 3.2. Domain 1—Physical Health

The statistical analysis proved a significant main effect for sex, *F*(1.491) = 5.58; *p* = 0.019; η_p_^2^ = 0.01. In women (*M =* 13.69; *SE =* 0.14) the quality of life in the somatic domain was lower than in men (*M =* 14.22; *SE =* 0.23).

The main effect for the field of study turned out not to be significant either, *F*(4.491) = 2.06; *p* = 0.085; η_p_^2^ = 0.02. Students of the technical university (*M =* 13.60; *SE =* 0.26), medicine (*M =* 14.10; *SE =* 0.29), social sciences (*M =* 14.25; *SE =* 0.26), obstetrics (*M =* 14.17; *SE =* 0.26), and dentistry (*M =* 13.63; *SE =* 0.29) do not differ in quality of life in the physical domain.

The interactive effect of both variables was significant, *F*(3.491) = 5.26; *p* = 0.001; η_p_^2^ = 0.03. The analysis of the simple effects for sex presented statistically significant differences between women and men of technical university, *F*(1.491) = 14.31; *p* < 0.001; η_p_^2^ = 0.03, and studying social sciences, *F*(1.491) = 7.99; *p* = 0.005; η_p_^2^ = 0.02. In both mentioned groups women were characterized with a lower quality of life than men. For both medicine, *F*(1.491) = 1.01; *p* = 0.316; η_p_^2^ < 0.01, and dentistry students, *F*(1.491) = 0.21; *p* = 0.649; η_p_^2^ < 0.01, the differences with regard to sex were not statistically significant.

**Figure 1:**
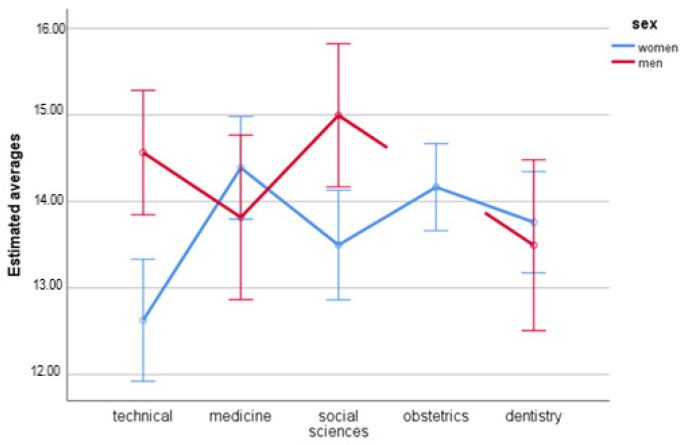
Estimated averages for first domain with regard to sex and field of study.

The analysis of simple effects with regard to the field of study presented statistically significant results only in women, *F*(4.491) = 4.36; *p* = 0.002; η_p_^2^ = 0.03. Female students of the technical university had a lower quality of life than women studying medicine (*p* = 0.002) and obstetrics (*p* = 0.005). That effect was not statistically significant in men, *F*(4.491) = 2.27; *p* = 0.080; η_p_^2^ = 0.01 (Table 3, Figure 1).

### 3.3. Domain 2—Psychological Health

No significant results were observed in the analysis of the main effects for sex, *F*(1.491) = 0.32; *p* = 0.574; η_p_^2^ < 0.01. In women (*M =* 13.60; *SE =* 0.16) and men (*M =* 13.67; *SE =* 0.26) the quality of life in the second domain was similar.

The main effect for the field of study turned out not to be significant either, *F*(4.491) = 1.45; *p* = 0.217; η_p_^2^ = 0.01. There is no significant differences between technical university students (*M =* 13.27; *SE =* 0.30), medical students (*M =* 13.46; *SE =* 0.34), social sciences students (*M =* 14.03; *SE =* 0.31), obstetrics students (*M =* 14.07; *SE =* 0.30), and dentistry students (*M =* 13.54; *SE =* 0.34) with regard to quality of life in the second domain.

There were significant differences when analyzing the interactive effect of both variables, *F*(3.491) = 2.87; *p* = 0.036; η_p_^2^ = 0.02. The analysis of simple effects for sex proved statistically significant differences between men and women in technical studies *F*(1.491) = 4.38; *p* = 0.037; η_p_^2^ = 0.01; women were characterized with a lower quality of life in the psychological domain than men. No differences with regard to sex were observed in students of social sciences, *F*(1,491) = 1.01; *p* = 0.316; η_p_^2^ < 0.01, medicine, *F*(1,491) = 3.80; *p* = 0.052; η_p_^2^ = 0.01, and dentistry, *F*(1.491) = 0.05; *p* = 0.831; η_p_^2^ < 0.01.

**Figure 2:**
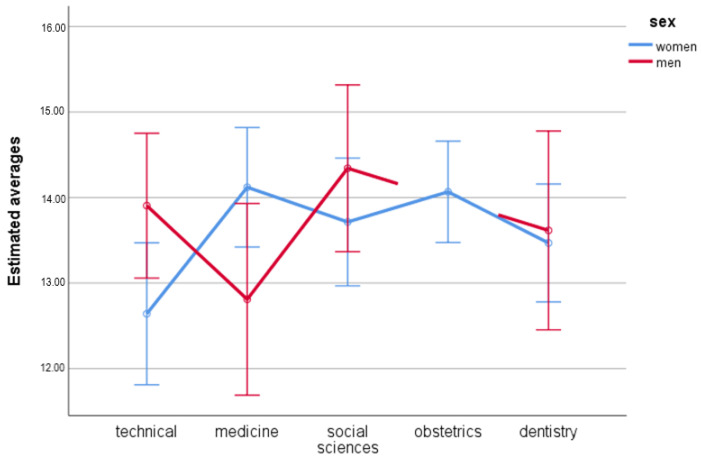
Estimated averages for second domain with regard to sex and field of study.

The analysis of simple effects for the field of studies showed statistically significant results only in women, *F*(4.491) = 2.40; *p* = 0.049; η_p_^2^ = 0.02. After the Benferroni correction was done, the effect turned out not to be significant. There were no differences between the students from different universities. In men, the effect was not significant, *F*(4.491) = 1.44; *p* = 0.230; η_p_^2^ = 0.01 (Table 4, Figure 2).

### 3.4. Domain 3—Social Relationships

The statistical analysis proved that there was no difference with regard to sex, *F*(1.491) = 0.02; *p* = 0.881; η_p_^2^ < 0.01. It can be stated that the quality of life in the third domain was similar in women (*M =* 14.51; *SE =* 0.18) and men (*M =* 14.33; *SE =* 0.29).

There was no statistically significant difference with regard to fields of study, *F*(4,491) = 1.34; *p* = 0.254; η_p_^2^ = 0.01. Technical university students (*M =* 14.20; *SE =* 0.33), medicine (*M =* 13.94; *SE =* 0.36), social sciences (*M =* 14.67; *SE =* 0.34), obstetrics (*M =* 15.00; *SE =* 0.33), and dentistry (*M =* 14.63; *SE =* 0.37) do not differ with regard to quality of life in third domain.

**Figure 3:**
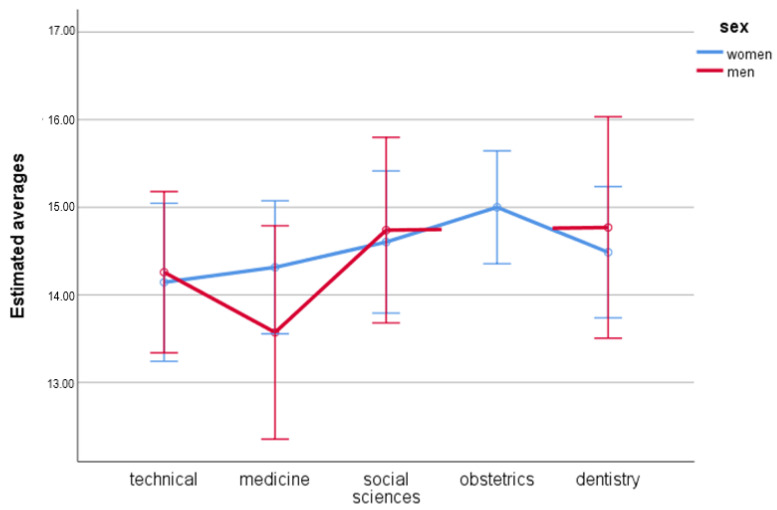
Estimated averages for the third domain with regard to sex and field of study.

There was no statistically significant difference when both variables were correlated (sex and fields of study), *F*(3.491) = 0.41; *p* = 0.744; η_p_^2^ < 0.01 (Figure 3).

### 3.5. Domain 4—Environment

The analysis of the main effect for sex showed no statistically significant differences, *F*(1.491) = 0.47; *p* = 0.493; η_p_^2^ < 0.01 in women (*M =* 14.03; *SE =* 0.13) and men (*M =* 14.15; *SE =* 0.20) the quality of life in the fourth domain was similar.

No difference was observed with regard to the fields of study, *F*(4.491) = 0.30; *p* = 0.880; η_p_^2^ < 0.01. Technical university students (*M =* 13.94; *SE =* 0.23), medicine (*M =* 14.10; *SE =* 0.26), social sciences (*M =* 14.13; *SE =* 0.24), obstetrics (*M =* 14.23; *SE =* 0.23), and dentistry (*M =* 14,08; *SE =* 0,27) showed no difference in quality of life in the fourth domain.

**Figure 4:**
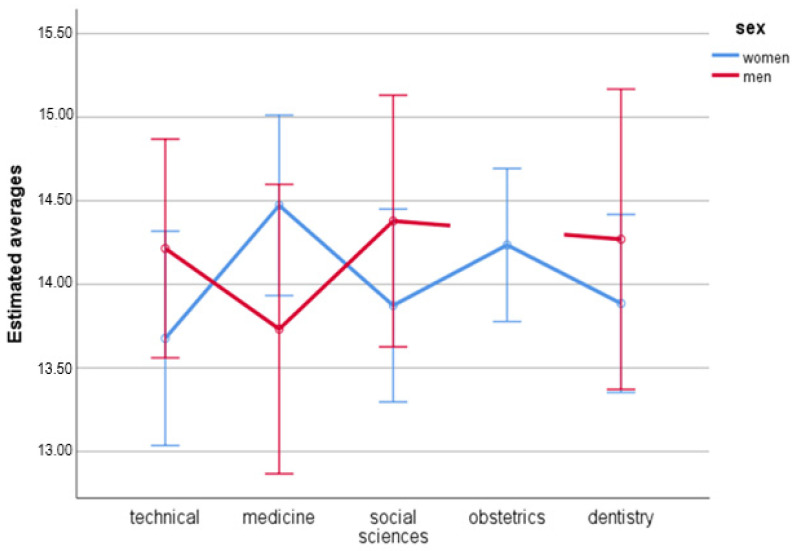
Estimates averages for the fourth domain with regard to sex and field of study.

No statistically significant differences were observed either when two variables (sex and field of studies) were correlated, *F*(3.491) = 1.44; *p* = 0.230; η_p_^2^ = 0.01 (Figure 4).

## 4. Discussion

The study was designed to review the concerns arising from the challenges that students were facing due to the COVID-19 pandemic with regard to the negative effects of pandemic on their psychophysical health conditions, by providing a brief, valid, and meaningful tool, namely the WHOQOL-BREF questionnaire to measure differences in quality of life of students from various faculties with regard to their level of medical knowledge. In the literature there is research available about the psychological impact of the epidemic on the general population, patients, health workers, children, and older adults. We can also find studies where the level of anxiety of university nursing students during the COVID-19 outbreak was discussed [17,18,19,20].

The WHOQOL-BREF questionnaire is successfully used by researchers from different areas to analyze the nature of COVID-19 related stressors that might be encountered by students as the obtained answers might be crucial to define tailored policies and support interventions.

The pandemic has strongly affected many sectors but undoubtedly mostly the health sector. Initially, healthcare students were under great pressure and had to deal with many uncertainties about the nature of the new disease and the implementation of brand new and very rigorous protocols. The stressors that the health students had to deal with were similar to those that doctors were exposed to. Undoubtedly, that strongly affected their ability to learn and caused an even higher level of anxiety [20,21,22].

The obtained results showed differences in respondents’ reactions in two domains. The lowest resistance to the critical situations, such as COVID-19, was observed in women who studied at the technical university. Higher values of resistance were observed in women studying medical sciences. Mocny-Pachońska et al., observed that first-year dental students were highly stressed. What is more, in that study it was noticed that women were associated with higher stress levels than men. Methods of coping with stressful situations, due to the cited study, were related to the sex of the participants. Using psychoactive substances and a sense of humor were typical for men, whereas women turned to religion and searched for instrumental and emotional support. She also reported that female students in more senior years were observed to have higher resistance to the extreme situation [23]. Higher levels of perceived stress among women were probably a result of comparing them to men (women are regarded to be more prone to physical and emotional problems such as depression and fatigue) [24]. Moreover, she observed that marital status strongly influenced stress development. In the case of single participants it was proved that they became more stressful when dealing with any situations that might influence their future careers.

Medical students are very often looked up to as the models of society. It needs to be remembered that they will become a very important part of the health system. The specific situation that all of us had to deal with during the times of the COVID-19 crisis and lockdown was an additional stress for medical students. There are several factors that influence the individual’s quality of life. These are health, changes to lifestyle, well-being, mental health, and life satisfaction. Very often it happens that any problems related to the mental health of medical students are neglected. During the COVID-19 pandemic that particular group of society had to deal not only with the disturbance of their daily routine but had also closely observed the work of other medical professionals who were fighting against a deadly and unknown virus [25]. In their studies, Rogowska et al., examined 914 students from Opole University of Technology, and assessed life satisfaction, general health, stress, and coping strategies during the COVID-19 pandemic. They observed a high level of perceived stress in 56% of participants. The variance of anxiety was influenced by following variables: sex, high stress, general health. In that research, the worst indices of mental health were observed in women than in men [21]. Their observations were similar to ours even though they observed students during the first lockdown and in our study it was the second wave of the coronavirus pandemic. 132 students from Polish universities were asked to fill in a questionnaire regarding the daily activities and impact of the necessity of keeping social distance in studies conducted by Szczepańska et al. [22]. They observed that 98% of respondents complained about the restrictions that needed to be followed and confirmed the decline in their mood and their quality of life. The WHOQOL-BREF questionnaire could be useful to early identify those students in need of psychological support. The adoption of the WHOQOL-BREF questionnaire in the clinical practice can significantly help social and health practitioners, serving as a monitoring and evaluation tool to define more tailored evidence-based counseling interventions.

### Limitaions of the Researsh

The realization of the aim of the study with the usage of an on-line questionnaire may limit the number of participants if any of them are using the internet with the restrictions. Our study was designed for students who are familiar with the internet as a method of communication, however it might be problematic in the cases of other respondents (for instance elderly people). Another limitation was the distribution of sex, which was caused by the specification of the field of studies we had chosen. Further research demands the increase of the number of participants (male) in order to prove the validity of presented results.

## 5. Conclusions

There were observed differences between women from technical and medical universities in the levels of resistance to critical situations, such as COVID-19. Higher values of resistance were observed in women studying medical sciences. These observations may lead to the conclusion that medical education and female sex might be a factor influencing higher resistance to stressful situations. The use of the questionnaire may be helpful to detect students who demand psychological consultation during the course of study, to define the stressors, and to organize tools to support students in need.

## Figures and Tables

**Table 1:** Characteristic of students involved in the study.

Medical University of Silesia	Dentistry	100
	Medicine	100
	Obstetrics	100
Maritime University of Szczecin	Navigation; Geodesy and Cartography	100
Adam Mickiewicz University, Poznań	Faculty of Political Science and Journalism	100

**Table 2:** Descriptive statistics with the test for normality of distribution and reliability of the domains.

Domain	M	Me	SD	Sk	Kurt	Min	Max	D	*p*	α Cronbach
Physical health	13.94	14.29	2.61	−0.46	−0.20	5.14	19.43	0.09	<0.001	0.762
Psychological health	13.71	14.00	3.04	−0.64	−0.05	4.00	20.00	0.11	<0.001	0.850
Social relationships	14.50	14.67	3.27	−0.56	0.11	4.00	20.00	0.13	<0.001	0.675
Environment	14.10	14.50	2.33	−0.59	0.28	5.00	19.00	0.11	<0.001	0.758

**Table 3:** Descriptive statistics for somatic domain with regard to sex and field of the studies.

Sex	Field of Studies	M	SE	95% CI	
				LL	UL
Women	technical (*n* = 51)	12.63	0.36	11.92	13.33
	medicine (*n* = 72)	14.39	0.30	13.80	14.98
	social sciences (*n* = 64)	13.50	0.32	12.86	14.13
	obstetrics (*n* = 100)	14.17	0.26	13.66	14.67
	dentistry (*n* = 74)	13.76	0.30	13.18	14.35
Men	technical (*n* = 49)	14.57	0.37	13.85	15.28
	medicine (*n* = 28)	13.82	0.48	12.87	14.77
	social sciences (*n* = 36)	15.00	0.42	14.17	15.82
	obstetrics (*n* = 0)	-	-	-	-
	dentistry (*n* = 26)	13.50	0.50	12.51	14.48

**Table 4:** Descriptive statistics for psychological health domain with regard to sex and field of studies.

Sex	Field of Studies	M	SE	95% CI	
				LL	UL
Women	technical (*n* = 51)	12.64	0.42	11.81	13.47
	medicine (*n* = 72)	14.12	0.36	13.42	14.82
	social sciences (*n* = 64)	13.71	0.38	12.97	14.46
	obestrics (*n* = 100)	14.07	0.30	13.47	14.66
	dentistry (*n* = 74)	13.47	0.35	12.78	14.16
Men	technical (*n* = 49)	13.90	0.43	13.06	14.75
	medicine (*n* = 28)	12.81	0.57	11.69	13.93
	social sciences (*n* = 36)	14.34	0.50	13.37	15.32
	obestrics (*n* = 0)	-	-	-	-
	dentistry (*n* = 26)	13.62	0.59	12.45	14.78

## Data Availability

The data presented in this study are available on request from the corresponding author.
